# Heavy Metal Induced Antibiotic Resistance in Bacterium LSJC7

**DOI:** 10.3390/ijms161023390

**Published:** 2015-09-29

**Authors:** Songcan Chen, Xiaomin Li, Guoxin Sun, Yingjiao Zhang, Jianqiang Su, Jun Ye

**Affiliations:** 1 State Key Lab of Urban and Regional Ecology, Research Center for Eco-Environmental Sciences, Chinese Academy of Sciences, Beijing 100085, China; E-Mails: scchen@iue.ac.cn (S.C.); xmli2013_st@rcees.ac.cn (X.L.); 2 Key Lab of Urban Environment and Health, Institute of Urban Environment, Chinese Academy of Sciences, Xiamen 361021, China; E-Mails: yjzhang@iue.ac.cn (Y.Z.); jqsu@iue.ac.cn (J.S.)

**Keywords:** LSJC7, antibiotic, heavy metal, resistance

## Abstract

Co-contamination of antibiotics and heavy metals prevails in the environment, and may play an important role in disseminating bacterial antibiotic resistance, but the selective effects of heavy metals on bacterial antibiotic resistance is largely unclear. To investigate this, the effects of heavy metals on antibiotic resistance were studied in a genome-sequenced bacterium, LSJC7. The results showed that the presence of arsenate, copper, and zinc were implicated in fortifying the resistance of LSJC7 towards tetracycline. The concentrations of heavy metals required to induce antibiotic resistance, *i.e.*, the minimum heavy metal concentrations (MHCs), were far below (up to 64-fold) the minimum inhibition concentrations (MIC) of LSJC7. This finding indicates that the relatively low heavy metal levels in polluted environments and in treated humans and animals might be sufficient to induce bacterial antibiotic resistance. In addition, heavy metal induced antibiotic resistance was also observed for a combination of arsenate and chloramphenicol in LSJC7, and copper/zinc and tetracycline in antibiotic susceptible strain *Escherichia coli* DH5α. Overall, this study implies that heavy metal induced antibiotic resistance might be ubiquitous among various microbial species and suggests that it might play a role in the emergence and spread of antibiotic resistance in metal and antibiotic co-contaminated environments.

## 1. Introduction

Co-existence of heavy metals and antibiotics occurs in many kinds of environmental matrices, such as gastrointestinal tracts, animal manure and poultry farm sites [1,2,3]. For example, arsenic and antibiotics are commonly used as food supplements in the livestock industry for disease control and growth promotion [4], which makes the intestinal microbiota of the domestic animals co-exposed to arsenic and antibiotics [3]. Furthermore, the application of poultry manure as fertilizer to the soil, the desired practice for recycling nutrients [5,6], causes the indigenous microorganisms to be exposed to arsenic as well as antibiotics [7]. Heavy metals are persistent in nature, accumulating in different components of the ecosystems [8]. Although many antibiotics have relatively short half-lives, they are regarded as “pseudopersistent” due to their continuous introduction into the ecosystem [5,9]. Such mixed contamination is causing considerable concerns, *i.e.*, whether their effects are combined with regard to selective ability of antibiotic resistance is unclear [10,11].

In some natural environments with microbial communities, combined contaminations of heavy metals and antibiotics contribute to the occurrence and spread of microbial antibiotic resistance; and sometimes multidrug resistance evolves [12]. For example, co-exposure to Zn and antibiotics such as oxytetracycline in activated sludge bioreactors appears to improve the resistance of the microbial community towards antibiotics [13]. The amendment of Cu in agricultural soils selects for Cu resistance and further co-selects for resistance to ampicillin, chloramphenicol and tetracycline [14]. Both Ni and Cd increased the frequency of bacterial resistance in microcosms to chemically unrelated antibiotics including ampicillin or chloramphenicol [15]. One possible explanation of such improvement of antibiotic resistance is that the presence of heavy metals enhanced the enrichment and growth of indigenous bacteria in the microbial community, which are already bearing antibiotic resistance genes; another possibility is that the resistance in bacteria which is sensitive to antibiotics could be induced due to the co-existence of heavy metals and antibiotics in the environment. Some investigations have demonstrated the positive correlation between the abundance of antibiotic resistance genes and the elevated concentrations of antibiotic and heavy metals in environments [2,16]. However, few reports are available about whether heavy metals could enhance the antibiotic resistance in the resistant bacteria or induce antibiotic resistance in sensitive bacteria [1].

Strain LSJC7, a Gram-negative member of the family *Enterobacteriaceae* in the order *Enterobacteriales* of the class *Gammaproteobacteria*, with dual resistance to arsenic and tetracycline, was previously isolated from an antimony tailing in China [17]. The whole genome of LSJC7 has been sequenced (AMFN00000000). This study systematically investigated the selective effects of heavy metal on antibiotic resistance in this strain, suggesting that bacterial antibiotic resistance could be induced by heavy metals.

## 2. Results and Discussion

### 2.1. Metal(loid) Resistance of LSJC7 and Identification of Putative Resistance Genes

**Figure 1:**
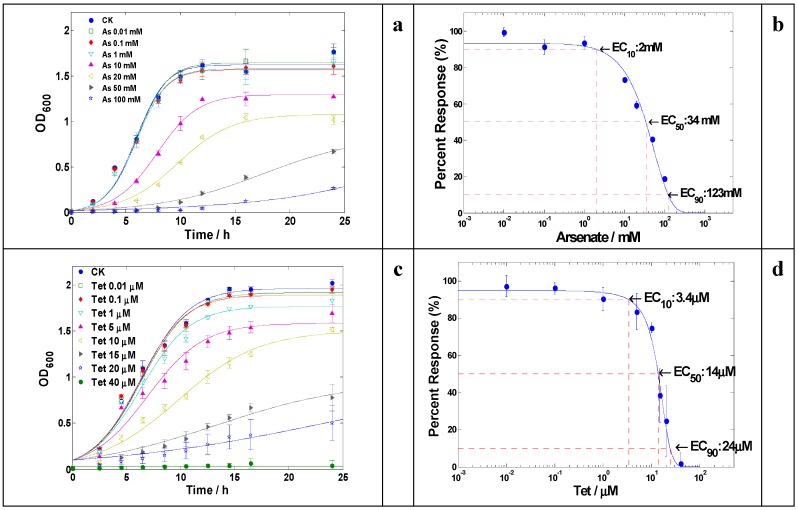
(**a**) Growth curves of LSJC7 with arsenate treatment; (**b**) Dose–response curve of LSJC7 with arsenate treatment; (**c**) Growth curves of LSJC7 with tetracycline treatment; (**d**) Dose–response curve of LSJC7 with tetracycline treatment.

The resistance of LSJC7 to arsenate was at a very high level, up to 100 mM in Luria-Bertani (LB) medium (Figure 1a); and the effective concentration of arsenate that caused a 90% of maximal growth inhibition (EC_90_) was estimated to be 123 mM (Figure 1b). The resistance of LSJC7 to arsenate was considerably higher than the reported arsenate resistant bacterium *Geobacillus kaustophilus* whose growth was completely prevented in the presence of 80 mM arsenate in LB broth [18]. Such high level of arsenate resistance in LSJC7 could be due to two arsenical resistance gene clusters (*arsRDABC* and *arsRBC*) in its bacterial chromosome (Table S1), which conferred arsenate resistance through arsenate reduction (arsenate reductase, ArsC) and active arsenite efflux (ArsB). Previous studies reported that bacteria expressing the *arsRDABC* operon were usually more resistant to arsenate than those expressing *arsRBC* operons because protein ArsA could form a complex with ArsB that catalyzes ATP-driven arsenite efflux [19], this might explain why LSJC7 possesses higher resistance to arsenate than *G. kaustophilus* which only contains *arsRBC*.

**Figure 2:**
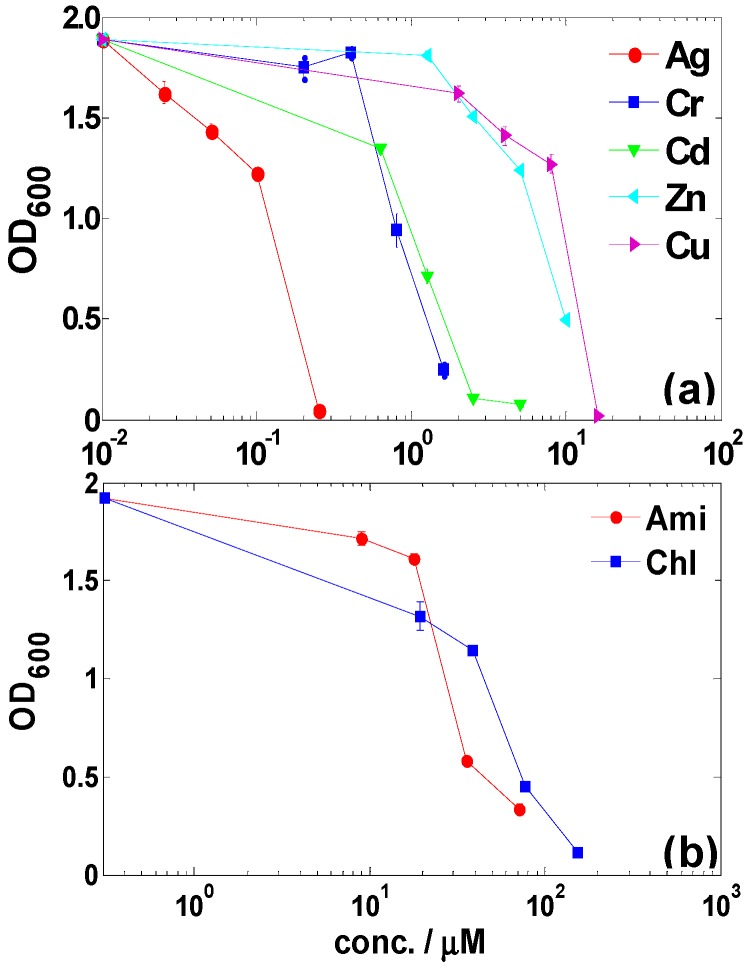
(**a**) Dose–response inhibitions of heavy metals on the growth of LSJC7; (**b**) Dose–response inhibitions of antibiotics on the growth of LSJC7. Ampicillin (Amp), chloramphenicol (Chl).

The resistance of LSJC7 towards other metals and metalloids were tested as well in this study. LSJC7 exhibited the highest resistance to Cu^2+^, while Ag^+^ was the most toxic compared to the other metal ions (Figure 2a). The minimum inhibition concentrations (MICs) for Cu^2+^, Zn^2+^, Cd^2+^, Cr_2_O_7_^2−^, and Ag^+^ were 16, 10, 2.5, 1.6 and 0.25 mM in LB medium, respectively. Various genes related to Cu^2+^, Zn^2+^, Cd^2+^, and Cr_2_O_7_^2−^ resistances have been identified by analyzing annotated genome of LSJC7 (Table S1).

### 2.2. Antibiotic Resistance of LSJC7 and Identification of Putative Resistance Genes

Tetracycline resistance of LSJC7 was tested in this study. During the incubation period, LSJC7 could grow in the presence of 20 μM (approx. 10 μg·mL^−1^) tetracycline, while no viable cells were recovered at concentrations more than 40 μM (Figure 1c); The calculated EC_10_, EC_50_, and EC_90_ values of tetracycline for LSJC7 were 3.4 μM, 14 μM, and 24 μM, respectively (Figure 1d). Analysis of the annotated genome of LSJC7 revealed the presence of putative genes responsible for multiple layers of tetracycline resistance systems, including ribosomal protection protein (*tetm*, *tetpb*, *otra*), tetracycline efflux pump (*otrb*, *tet39*, *tetb*), and tetracycline modification enzyme (*tet34*)-like genes.

The resistance of LSJC7 to chloramphenicol and ampicillin was also evaluated. Results showed that the MICs of LSJC7 for chloramphenicol and ampicillin were slightly lower than 155 μM (approximately 50 μg·mL^−1^) and 72 μM (approximately 25 μg·mL^−1^), respectively (Figure 2b). As LSJC7 exhibited resistant to three classes of antimicrobial agents, it could be referred to as multi-resistant according to the definition given by Schwarz *et al.* [20]. Further analysis based on genomic inspection revealed that the multi-resistance phenotype of LSJC7 may be ascribed to the presence of specific resistance genes and multidrug resistance (MDR) determinants (e.g., multidrug efflux transporter) in the chromosome (Table S2). The broadness of antibiotic resistome in LSJC7 might be a reflection of powerful selection pressure in the environment such as antibiotics or even heavy metals [12].

### 2.3. Enhancement of Antibiotic Resistance in the Presence of Heavy Metals

**Figure 3:**
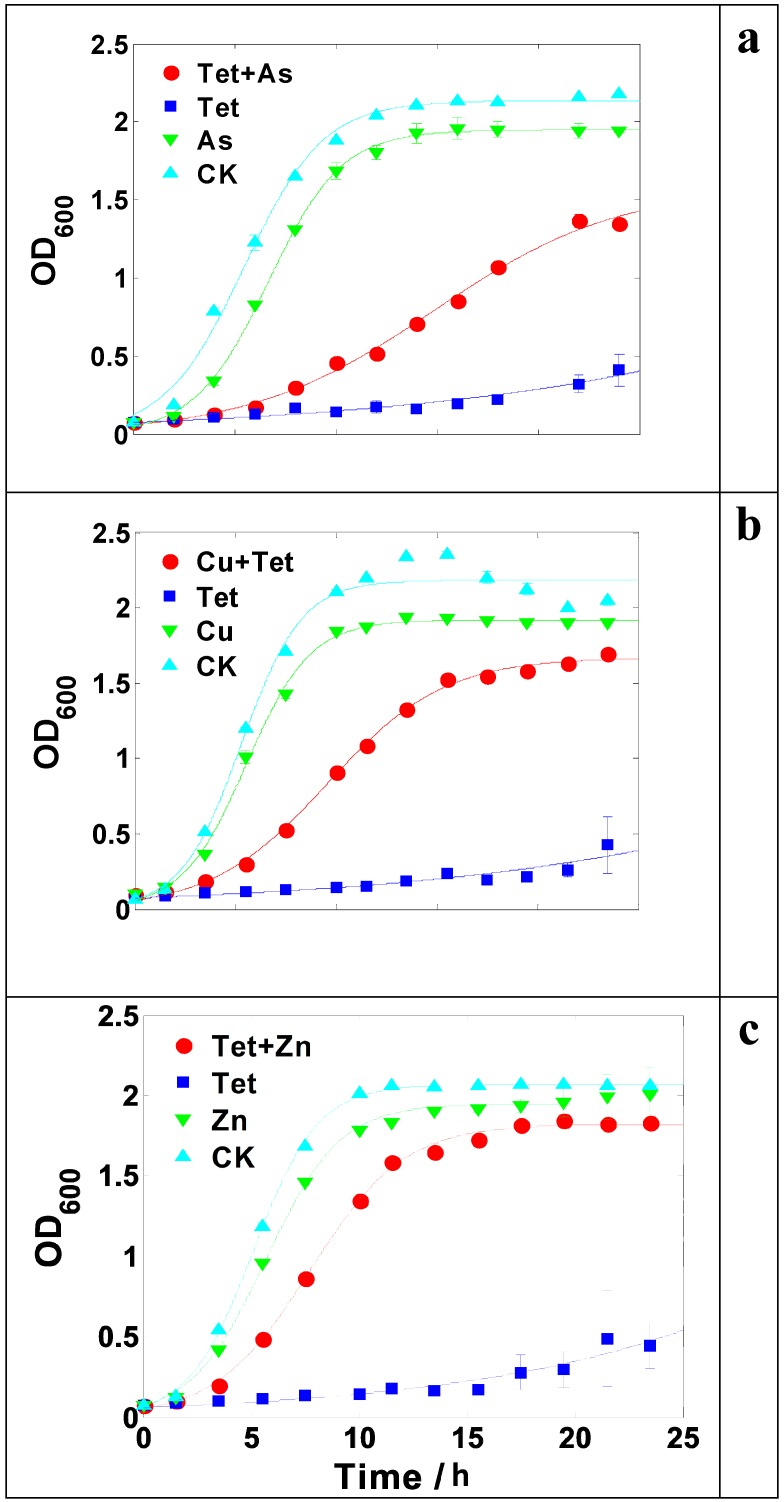
(**a**) Growth curve of LSCJ7 with 2 mM arsenate (As) and/or 24 μM tetracycline (Tet) treatment; (**b**) Growth curve of LSJC7 with 4 mM copper (Cu) and/or 24 μM Tet treatment; (**c**) Growth curve of LSCJ7 with 1.25 mM zinc (Zn) and/or 24 μM Tet treatment. Each point is presented as mean ± SD (*n* = 3). Growth curves are fitted by logistic model.

**Figure 4:**
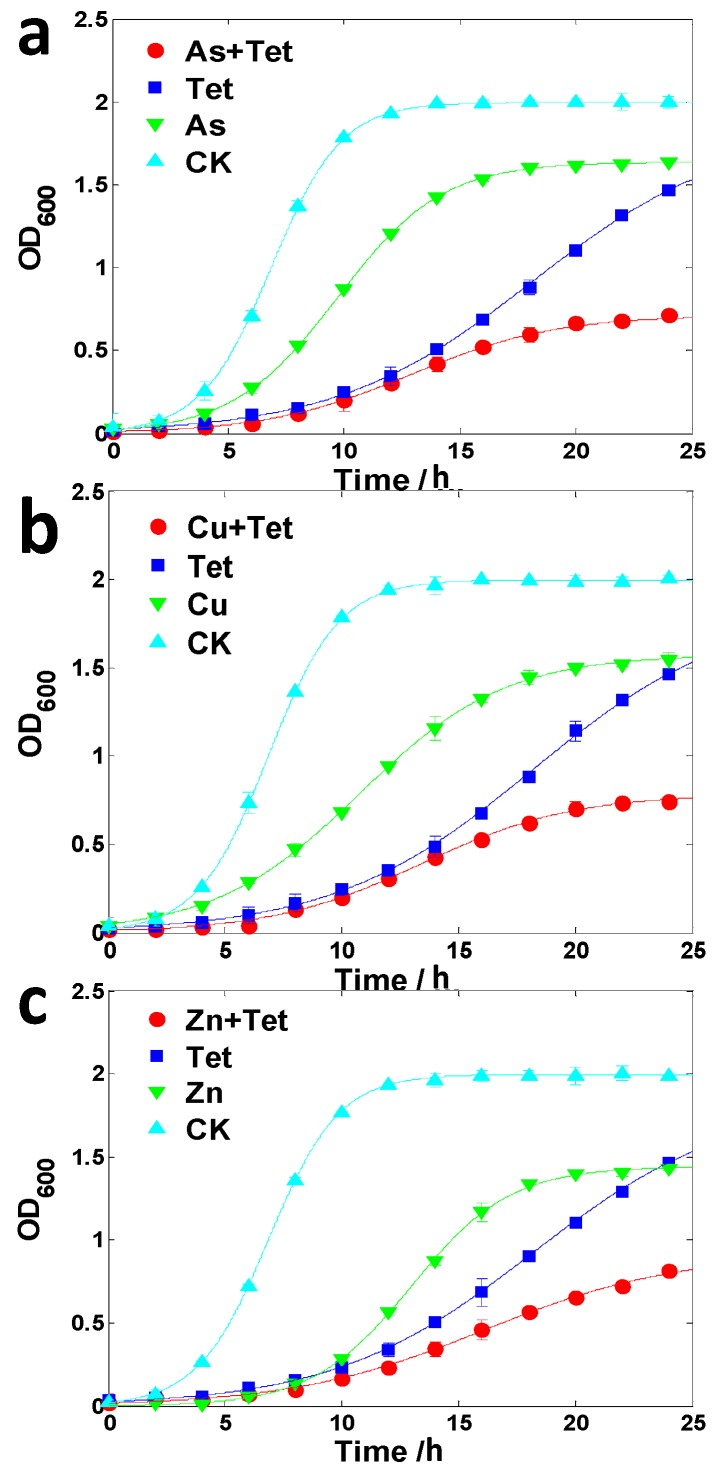
(**a**) Growth curve of *Pseudomonas oryzihabitans* with 2 mM arsenate (As) and/or 2 μM tetracycline (Tet) treatment; (**b**) Growth curve of *P. oryzihabitans* with 4 mM copper (Cu) and/or 2 μM Tet treatment; (**c**) Growth curve of *P. oryzihabitans* with 5 mM zinc (Zn) and/or 2 μM Tet treatment. Each point is presented as mean ± SD (*n* = 3). Growth curves are fitted by logistic model.

The growth of LSCJ7 was significantly promoted in the presence of arsenate (2 mM) and tetracycline (24 μM) compared to the equivalent tetracycline treatment alone (Figure 3a), indicating that the existence of arsenate enhanced bacterial tetracycline resistance. Similarly, copper (4 mM) or zinc (1.25 mM) could also increase tetracycline resistance in LSJC7 (Figure 3b,c). In contrast, the resistance of *Pseudomonas oryzihabitans* to tetracycline turned out to be decreased by the addition of arsenate, zinc, or copper (Figure 4).

Heavy metal induced tetracycline resistance should be paid more attention. The concentration used here corresponds to 150 μg·mL^−1^ arsenic and 12 μg·mL^−1^ tetracycline, a level that has been found in many types of environments [21,22]. For example, both arsenic compound and tetracycline have been commonly used in large quantities as growth promotion agents in animal feed, mainly as roxarsone and p-arsanilic acid [23]. The level of organoarsenic and tetracycline in swine feed can be as high as 100 and 40 mg·kg^−1^, respectively [6,24], which corresponds to a molar amount of arsenic and tetracycline approximately equal to the concentration used in this study. Thus, microbes present in animal intestines might be better equipped to survive under tetracycline pressure with the presence of arsenic, and hence have more opportunity to develop further mutations in genes encoding antibiotics resistance [25]. Furthermore, most of the organoarsenic and tetracycline are poorly absorbed by animals [23,26,27], and manure collected from feedlots has been shown to contain approximately 12 mg·kg^−1^ of tetracycline and 110 mg·kg^−1^ of arsenic coincidently [28]. With the common practice of using poultry and swine manure as fertilizers on agricultural lands, potentially significant amount of arsenic and tetracycline can be released into the soils and water together [29,30,31]. Moreover, copper or zinc could also increase tetracycline resistance in LSJC7 (Figure 3). This is problematic since these two kinds of metals are also widely applied in industries, agriculture, constructions, healthcare, and other areas from which they are released together with antibiotics into the environment [32,33].

**Figure 5:**
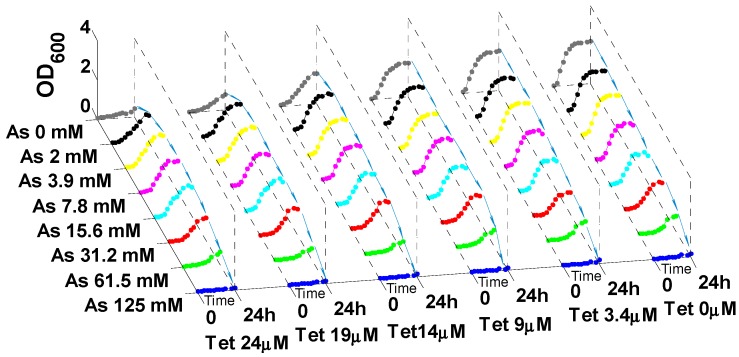
Growth curve of LSJC7 with tetracycline (Tet) and arsenate (As) co-treatment.

Different co-exposure levels of heavy metal and antibiotic were tested to identify the dose combinations for heavy metal induced antibiotic resistance in LSJC7. The results showed low-metal and high-antibiotic concentrations were prerequisite for heavy metal induced antibiotic resistance (Figure 5, Figures S1 and S2). (i) High levels (>EC_50_) of antibiotic were required for heavy metal to exert stimulating effects on antibiotic resistance. As shown in Figure 5, at exposure equal or lower than 9 μM tetracycline (EC_50_), the growth of LSJC7 was steadily reduced with increasing arsenate (copper or zinc) concentrations, which indicated that arsenic (copper or zinc) application could not enhance the resistance of LSJC7 to lower concentrations of tetracycline; (ii) Sub-toxic levels of heavy metal (<EC_50_) were able to induce antibiotic resistance in LSJC7. Results showed that the minimum heavy metal concentrations (MHC) to induce antibiotic resistance in LSJC7 were significantly below the corresponding MICs (Figure 5): for arsenic and zinc, the MHC value was 1/64 of the MIC; for copper, the value was 1/16. These values represent absolute heavy metal concentrations of 2 mM (arsenate), 1 mM (copper) and 0.16 mM (zinc). In contrast, when arsenate (copper or zinc) was imposed at lethal concentration, fortification of tetracycline resistance disappeared in LSJC7 (Figure 5). This disappearance of fortification might be due to the overall metabolic dysfunction in LSJC7 caused by excessive arsenate (copper or zinc), as higher concentrations of heavy metals are toxic to microbes [34].

The fact that the concentrations of heavy metals required to induce antibiotic resistance lie well below (16- to 64-fold) the concentration necessary to prevent growth of bacteria is of great concern [35], since it indicates that even relatively small amount of heavy metal in the human body, with the concentration up to MHCs, could challenge antibiotic therapies. The risks of accumulating heavy metal to MHC levels in human body can be relatively high in some areas of the world. For example, cadmium contents in 3.3% of rice samples were above maximum allowable concentration in China [36], where rice is the most important staple crop. Furthermore, there are large areas around the world where the groundwater naturally contains high levels of inorganic arsenic, such as in Bangladesh, where millions of people get their drinking water from wells containing up to 300 μg·L^−1^ [37]. In addition, some countries and areas, such as United States, Singapore, China and Hong Kong, use traditional Chinese medicine (TCM) as preferred medical treatment, of which heavy metal contamination has been frequently reported [38]. Thus, people in these areas are more likely to accumulate heavy metals, and hence might become less sensitive to antibiotic treatment because of the antibiotic resistance induced by heavy metals.

In addition to arsenate (copper or zinc) induced tetracycline resistance in LSJC7, other combinations of heavy metals and antibiotics showed similar effects on bacterial antibiotic resistance (Table 1). Firstly, arsenate was capable to fortify the antibiotic resistance in LSJC7 other than tetracycline. Figure S3a showed that arsenate at 4 mM could significantly alleviate the growth inhibition of chloramphenicol (77 μM) on LSJC7. Moreover, *Escherichia coli* DH5α, a susceptible strain to tetracycline, had its resistance enhanced by 2 mM copper or 0.625 mM zinc (Figure S3). Mallik *et al.* [39] reported that clinical strains of *Yersinia enterocolitica* biovar 1A exhibited multiple antibiotic resistance induced by arsenite. In *Pseudomonas aeruginosa*, copper and zinc treatments caused resistance not only to metals but also to carbapenem antibiotics [40]. *Escherichia hermannii* and *Enterobacter cloacae* were shown to possess multidrug resistance induced by vanadium [41]. In all, our observations, combined with other studies, showed a broad distribution of antibiotic resistance induced by heavy metal among various microbial species, and implied heavy metal induced antibiotic resistance might play a role in maintenance and dissemination of antibiotic resistance at the sites co-contaminated with heavy metals and antibiotics.

**Table 1:** Summery of heavy metal mediated antibiotic resistance in LSJC7 and *Escherichia coli* DH5α.

Strain	LSJC7			*E. coli* DH5α		
Combination	Tet	Amp	Chl	Tet	Amp	Chl
As	*	/	*	/	/	/
Cu	*	/	/	*	/	/
Zn	*	/	/	*	/	/

* indicates heavy metal mediated antibiotic resistance existed between two antimicrobial agents; / indicates heavy metal mediated antibiotic resistance did not exist between two antimicrobial agents.

### 2.4. Possible Mechanisms of Heavy Metal Induced Antibiotic Resistance

The expression of antibiotic resistance system in bacteria up-regulated by heavy metals might be responsible for the heavy metal enhanced antibiotic resistance (co-regulation). Several genes responsible for antibiotic resistance in LSJC7 were significantly up-regulated in the presence of arsenate (unpublished data). For example, a multiple antibiotic resistance gene, *emrD* (LSJC7GL004282), was up-regulated 1.6-fold in co-exposure to arsenate and tetracycline compared with that exposed to tetracycline alone, which might facilitate LSJC7 with higher antibiotic resistance in the presence of arsenate. In addition, compared with control, the expression of *emrD* and a tetracycline resistance gene, *tet34* (LSJC7GL000047), were up-regulated by 3.8- and 2.7-fold, respectively, in the response to arsenate. These results suggested that the expression of antibiotic resistance genes could be modulated by arsenate, which subsequently increased the resistance to antibiotics.

**Figure 6:**
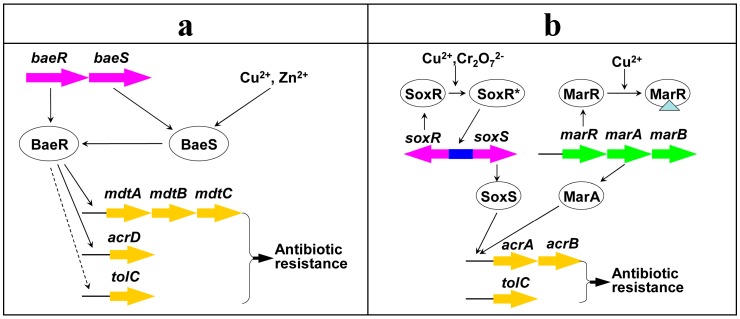
Possible mechanism (co-regulation) for heavy metal induced antibiotic resistance. (**a**) Pathway regulated by two-component signal transduction system BaeRS; (**b**) pathway regulated by global regulator SoxR or MarR. Identified or deduced pathways are represented as solid or dashed lines, respectively; the inducer of the repressor protein is represented as triangle; genes (gene clusters) are represented as colored arrows; * indicates the induced protein.

Consistently, several other studies showed that the expression of bacterial antibiotic resistance systems could be induced by some heavy metals, resulting in enhanced antibiotic resistance. For example, the transcription of multi-drug (antibiotic) efflux pump genes *acrD* and *mdtABC* in *Salmonella enterica*, can be induced by a two-component signal transduction system BaeRS in response to copper or zinc (Figure 6), causing increased bacterial antibiotic resistance [42]. The multi-drug efflux pump AcrAB-TolC in *E. coli* (Figure 6), conferring resistance to diverse antibiotics, could be up-regulated by a global regulator SoxS in response to chromate or copper [43], which led to enhanced tolerance toward antibiotics [44]. Copper acted as an inducer to derepress multiple antibiotic resistance regulatory operon *marRAB* in *E. coli* (Figure 6), causing enhanced bacterial antibiotic resistance [45]. As the genes mentioned above (*baeRS*, *acrD*, *mdtABC*, *soxS*, *acrAB*, *tolC*, *marRAB*) have been identified in the genome of LSJC7 (Table S3), we assumed that this regulatory network might provide a basic picture in controlling heavy metal induced antibiotic resistance in LSJC7 (Figure 6).

In addition, the chemical reactions between heavy metal and antibiotic might also lead to the phenomenon of bacterial cross-resistance. The effective concentrations of heavy metal and antibiotic may be affected by the chelation of antibiotics with metals to decrease the bioavailability of each other [46]. For example, the bioavailable tetracycline could be reduced by cationic ions such as copper (or zinc), due to the formation of complexes between metallic cations and tetracycline [47,48]. Therefore, the bacterial tetracycline resistance might be strengthened for the decreasing bioavailable concentration [48].

Antibiotic resistance induced by heavy metals reported in this study is alarming, although its exact mechanism remains unclear. In clinical settings, combination therapies involving silver and antibiotics have been suggested for treating Gram-negative infections [49]. However, our results indicated that precautions should be taken in such therapies, because co-treatment with heavy metals and antibiotics might induce antibiotic resistance in certain pathogenic bacteria and thus exacerbate infections [50]. In addition, combined use of heavy metals and antibiotics in intensive animal farming is still common and even unmonitored [2]. In such co-exposure systems, heavy metal induced antibiotic resistance might represent a novel antibiotic resistance mechanism [40] and could play a role in the maintenance and proliferation of antibiotic resistance reservoirs of clinically important microorganisms [12], which challenge life-saving antibiotic therapies [51].

## 3. Experimental Section

### 3.1. Bacterial Strains and Growth Medium

Strain LSJC7, a Gram-negative member of the family *Enterobacteriaceae*, was previously isolated in our laboratory. The strain was grown in LB medium (pH 7.0; 10 g NaCl, 5 g yeast extract, and 10 g tryptone in 1 L of double-distilled water).

### 3.2. Dose-Response Inhibition of Growth for Heavy Metals

Stock solutions of CuSO_4_·5H_2_O, ZnSO_4_·7H_2_O, CdCl_2_·2H_2_O, AgNO_3_, Na_3_AsO_4_, and K_2_Cr_2_O_7_ (Sinopharm Chemical, Zhonglian Chemical, Liaocheng, China) were prepared, respectively, in double-distilled water, passed through a 0.22 μm syringe filter and stored at 4 °C until use.

The stock solution of heavy metal or metalloid oxyanion was diluted in LB broth followed by adjustment of the pH to 7.0, which served as working solution. The following concentrations of metals were chosen for working solution: Cu^2+^, 16 mM; Zn^2+^, 10 mM; Cd^2+^, 5 mM; Ag^+^, 0.25 mM; Cr_2_O_7_^2−^, 1.6 mM; and AsO_4_^3−^, 100 mM. Serial twofold dilutions of the working solution were made in LB broth along the length of a 96-well microtiter plate (resulting in a serial concentration gradient from maximum concentration to minimum concentration). The first and last wells of every row were used as sterility and growth controls, respectively. Following 12 h exposure to heavy metals, the mean optical density at 600 nm (OD_600_) was measured by a microplate reader. Each heavy metal concentration was replicated in three wells.

### 3.3. Dose–Response Inhibition of Growth for Antibiotics

Stock solutions of tetracycline, ampicillin, or chloramphenicol (SolarBio, Beijing, China) were prepared in double-distilled water, passed through a 0.22 μm syringe filter and stored at −20 °C until use. The concentration of antibiotic added in LB broth as the working solution is 12, 25, and 50 μg·mL^−1^ for tetracycline, ampicillin, and chloramphenicol, respectively. The following experimental procedure was the same as that described in the experiment with heavy metal exposure.

### 3.4. Growth Kinetics of Bacteria

Bacteria were grown overnight in LB medium at 37 °C, diluted 1:100, and re-grown in 96-well microtiter plates containing 200 μL LB media with different concentrations of heavy metals and/or antibiotics in each well (the concentrations of heavy metal and antibiotic were chosen according to dose-response curve). Then these membrane-sealed microtiter plates were incubated at 30 °C on a shaker for 24 h with agitation of 200 rpm. The growth kinetics of the cells were monitored by measuring OD_600_ every two hours using a Thermomax microplate reader with SoftMax Pro data-analysis software (Molecular Devices, Sunnyvale, CA, USA). In order to calibrate the results measured by microplate reader to OD_600_ detected by Spectrometer (light path length was fixed to 1 cm), the linear relationship between microplate reader and Spectrometer has been established for data normalization.

### 3.5. Metal and Antibiotic Analysis

Inductively coupled plasma-optical emission spectrometry (ICP-OES; Optima 8300 DV (PerkinElmer, Waltham, MA, USA)) was used to determine the concentrations of As, Cd, Cu, Zn, Ag, and Cr in LB culture media without any bacterium at the initial (0 h) time. The aliquots (0.5 mL each) were diluted and acidified using 1% HNO_3_ to 50 mL in the tubes before measurement. The concentrations of tetracycline, ampicillin, and chloramphenicol in LB culture media were determined by liquid chromatography in combination with tandem mass spectrometry (LC-MS/MS; ABI 3200 Q TRAP (SCIEX, Arcade, NY, USA)), following described methods [52]. No significant differences were found between the nominal and measured exposure concentrations of heavy metals or antibiotics. Therefore, throughout the present study, nominal concentrations were used for data analysis.

### 3.6. Data Analysis

Dose-response inhibition of cells (treated with arsenate or tetracycline) and all growth kinetics were modeled by fitting a logistic function as shown in the following equation:

(1) $$y=\frac{a}{1+be^{-cx}}$$

where *y* represents OD_600_; *x* represents the concentration of antibiotics or growth time, in dose-response inhibition curve or growth kinetics curve, respectively; *a*, *b*, and *c* represent the regression coefficients. Data analysis and graphing of the experimental results were completed by EXCEL2003 and MATLAB2012a software.

## 4. Conclusions

In conclusion, this study revealed that the existence of heavy metals could enhance bacterial tetracycline resistance. Even heavy metals at sub-toxic levels were shown to induce bacterial antibiotic resistance, and such inducible antibiotic resistance might be ubiquitous among various microbial species in the environment. The fact that bacterial antibiotic resistance could be enhanced by heavy metals should be highlighted, as it might pose risks to environment and public health.

## Supplementary Materials

Supplementary materials can be found at http://www.mdpi.com/1422-0067/16/10/23390/s1.
