# Supplementary material for: Heavy Metal Induced Antibiotic Resistance in Bacterium LSJC7
Source: Int J Mol Sci. 2015 Sep 29;16(10):23390–404. doi: 10.3390/ijms161023390 (PMC4632705; doi:10.3390/ijms161023390)
Supplement: Supplementary file 1 [file ijms-16-23390-s001.pdf]

Supplementary Information

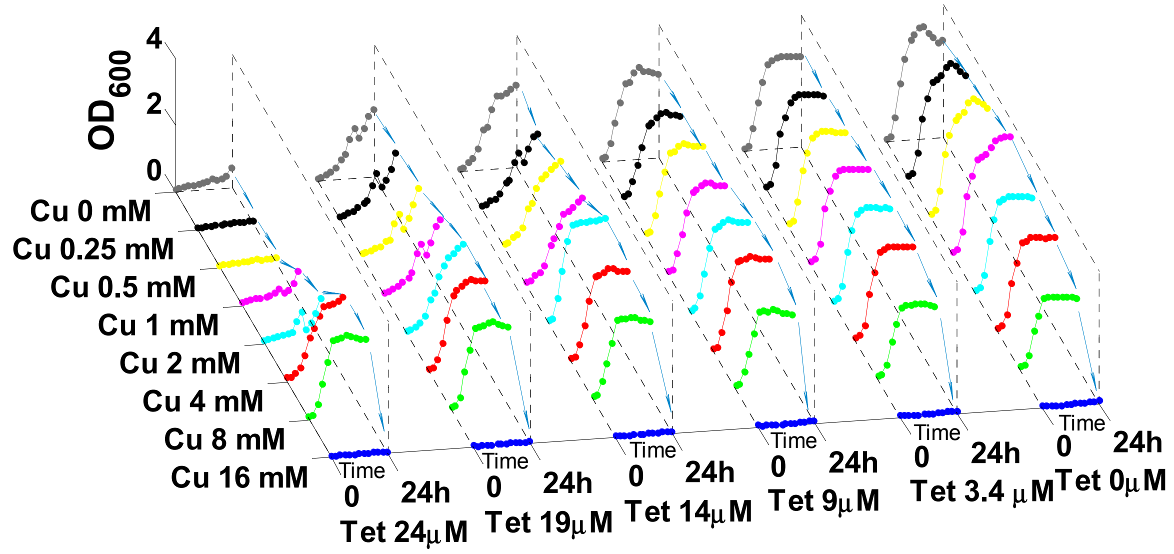

Figure S1. Growth curve of LSJC7 with tetracycline (Tet) and copper (Cu) co-treatment. Each point is presented as mean ± SD (n = 3).

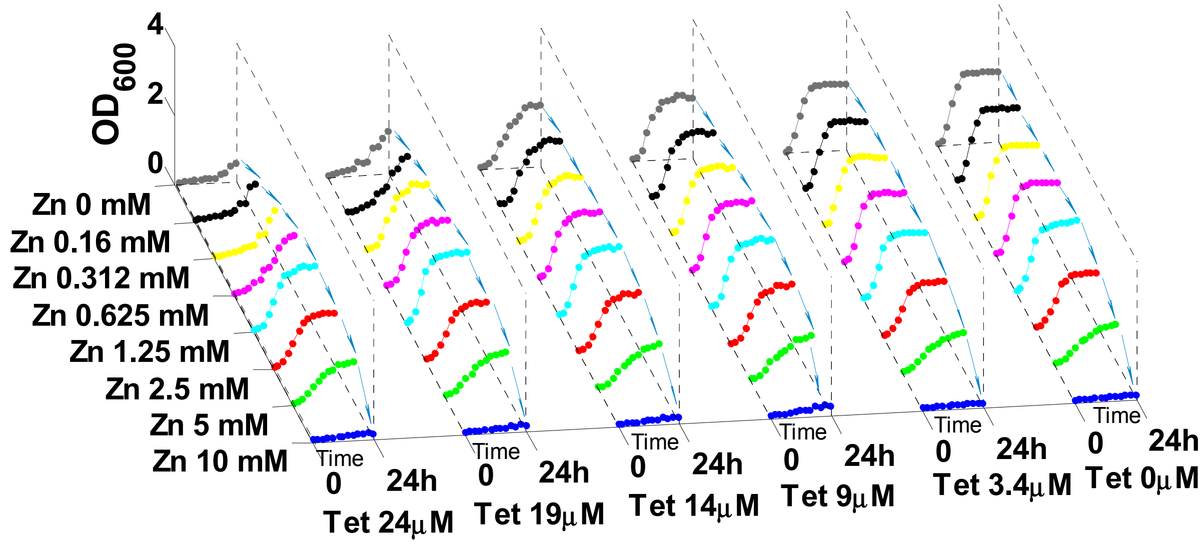

Figure S2. Growth curve of LSJC7 with tetracycline (Tet) and Zinc (Zn) co-treatment. Each point is presented as mean ± SD (n = 3).

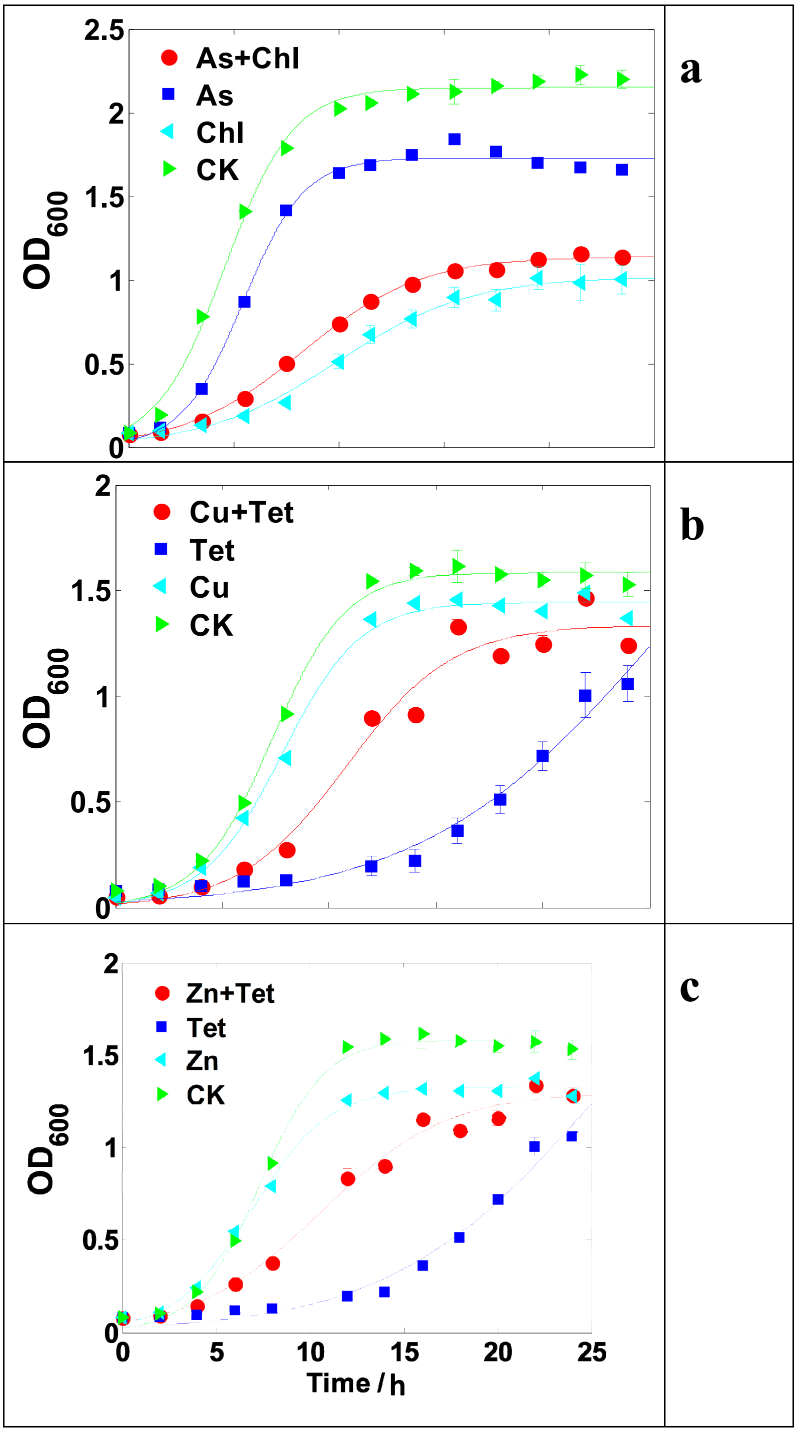

Figure S3. Growth curve of LSJC7 and *E. coli* DH5α with antibiotic and heavy metal co-treatment. (**a**) Growth curve of LSCJ7 with 4 mM arsenate (As) and 77 μM chloramphenicol (Chl) cotreatment; (**b**) Growth curve of *E. coli* DH5α with 2 mM copper (Cu) and 2 μM tetracycline (Tet) cotreatment; (**c**) Growth curve of *E. coli* DH5α with 0.625 mM zinc (Zn) and 2 μM Tet cotreatment. Each point is presented as mean ± SD (n = 3). Growth curves are fitted by logistic model.

Table S1. Genes involved in metal(loid) resistance that were identified in the draft genome of LSJC7.

Metal(loid)	Gene Locus_Tag	Function
Arsenic	LSJC7GL001421, LSJC7GL001422, LSJC7GL001423, LSJC7GL001424, LSJC7GL001425	Arsenical resistance gene cluster ArsRDABC
-	LSJC7GL002469, LSJC7GL002470, LSJC7GL002471	Arsenical resistance gene cluster ArsRBC
-	LSJC7GL002190, LSJC7GL002205,	Arsenate reductase ArsC
-	LSJC7GL001573	Arsenical pump membrane protein ArsB
Copper	LSJC7GL00191	Copper-transporting P-type ATPase CopA
-	LSJC7GL00881	Copper resistance protein CopC
-	LSJC7GL00882	Copper export protein P PcoD
Zinc	LSJC7GL000454	Zinc transporter zitB
-	LSJC7GL001091	Zinc transport protein ZntB
Cadmium	LSJC7GL003182	Cadmium, lead, zinc and mercury-transporting ATPase ZntA
Chromate	LSJC7GL001346	chromate reducatase
-	LSJC7GL004041	chromate reductase monomer

Table S2. Genes involved in antibiotic resistance that were identified in the draft genome of LSJC7.

Antibiotic	Gene Locus_Tag	Function
Tetracycline	LSJC7GL003015, LSJC7GL002387, LSJC7GL003618	Ribosomal protection protein TetM
-	LSJC7GL002366<br>LSJC7GL004289	Ribosomal protection protein Tetpb<br>Ribosomal protection protein Otra
-	LSJC7GL003544	Tetracycline efflux pump Otrb
-	LSJC7GL003544, LSJC7GL004005, LSJC7GL000161, LSJC7GL000896, LSJC7GL001857, LSJC7GL000962	Tetracycline resistance protein/ tetracycline efflux pump TetB
-	LSJC7GL003544	Tetracycline efflux pump Otrb
-	LSJC7GL002274	Tetracycline efflux pump Tet39
-	LSJC7GL000047, LSJC7GL002899	tetracycline modification enzyme tet34
Chloramphenico l	LSJC7GL001635<br>LSJC7GL000534	Chloramphenicol efflux pump Cml Multidrug/ chloramphenicol efflux transport protein MdfA
-	LSJC7GL001639	Bicyclomycin/chloramphenicol resistance protein YdhC
-	LSJC7GL001959	Bicyclomycin/chloramphenicol resistance protein Bcr
Ampicillin	LSJC7GL001204	Beta-lactamase ampC

Table S3. Genes involved in co-regulation system that were identified in the draft genome of LSJC7.

Efflux Pump	Pump Locus_Tag	Regulator	Regulator Locus_Tag	Inducible Signal	Reference
AcrD	LSJC7GL002188	BaeSR<br>CpxAR	LSJC7GL001858, LSJC7GL001859<br>LSJC7GL004139, LSJC7GL004140	Zinc, copper<br>Zinc, copper	[42]
MdtABC	LSJC7GL001854, LSJC7GL001855, LSJC7GL001856	BaeSR<br>CpxAR	LSJC7GL001858, LSJC7GL001859<br>LSJC7GL004139, LSJC7GL004140	Zinc, copper<br>Zinc, copper	[42]
AcrAB	LSJC7GL000169<br>LSJC7GL000170	MarRAB<br>SoxRS	LSJC7GL001469, LSJC7GL001470, LSJC7GL001471<br>LSJC7GL002479<br>LSJC7GL002480	Copper<br>Chromate, copper	[43,45]
